# Vehicular Visible Light Networks for Urban Mobile Crowd Sensing

**DOI:** 10.3390/s18041177

**Published:** 2018-04-12

**Authors:** Barbara M. Masini, Alessandro Bazzi, Alberto Zanella

**Affiliations:** CNR-IEIIT, v.le Risorgimento, 2, 40136 Bologna, Italy; alessandro.bazzi@ieiit.cnr.it (A.B.); alberto.zanella@ieiit.cnr.it (A.Z.)

**Keywords:** connected vehicles, vehicular networks, visible light communications, IEEE 802.11p, DSRC, complementary technologies, heterogeneous networks, crowd sensing, offloading

## Abstract

Crowd sensing is a powerful tool to map and predict interests and events. In the future, it could be boosted by an increasing number of connected vehicles sharing information and intentions. This will be made available by on board wireless connected devices able to continuously communicate with other vehicles and with the environment. Among the enabling technologies, visible light communication (VLC) represents a low cost solution in the short term. In spite of the fact that vehicular communications cannot rely on the sole VLC due to the limitation provided by the light which allows communications in visibility only, VLC can however be considered to complement other wireless communication technologies which could be overloaded in dense scenarios. In this paper we evaluate the performance of VLC connected vehicles when urban crowd sensing is addressed and we compare the performance of sole vehicular visible light networks with that of VLC as a complementary technology of IEEE 802.11p. Results, obtained through a realistic simulation tool taking into account both the roadmap constraints and the technologies protocols, help to understand when VLC provides the major improvement in terms of delivered data varying the number and position of RSUs and the FOV of the receiver.

## 1. Introduction

Vehicles are becoming sensing tools, paving the way to new challenging applications ranging from safety to environmental sensing and prediction of driver behaviors. A car today is equipped with over 4000 sensors that continuously collect internal and external data [1]. In addition, the presence of on board unit (OBUs) equipped with wireless communication technologies enables nearly real time transmissions of the collected information toward other road users and the network.

Among the wireless enabling technologies, we are assisting at the race between IEEE 802.11p and Cellular-V2X (C-V2X) to win the podium of vehicle-to-everything (V2X) communications [2,3]. On the one hand, IEEE 802.11p still represents the most tested and consolidated technology for vehicular communications and have shown good performance also in challenging situations [4]; on the other hand, C-V2X is based on a widely diffused network and provides higher performance, especially in crowded scenarios, and long term technical support by the ecosystem [5].

Independently on which one of these two radio frequency (RF) technologies will reach the maximum diffusion for on board integration in the next future, recent years also assisted to the increasing interest in visible light communication (VLC) to enable vehicular visible light networks [6,7,8,9]. Today vehicles are, in fact, already equipped with light emitting diode (LEDs) which can be easily modulated and exploited also for communication purposes. This way, vehicle-to-vehicle (V2V) communications can be achieved directly using the front and rear lights, without the effort of new deployment cost. In addition, since also most of road infrastructures are LED-based and traffic lights or variable message panel already cover most urban and suburban areas, also vehicle-to-infrastructure (V2I) communications could be enabled without the installation of expensive road side units (RSUs). However, VLC transmission is strongly subjected to the presence of obstacles and bad weather conditions over the optical channel, such as rain, snow and fog, which could cause poor or no visibility, making LED communications impractical.

Recent literature investigates the use of VLC in vehicular networks in terms of feasibility and in comparison or in addition to dedicated short-range communications (DSRC) [9]. As for sensors, where only one type is not often sufficient to achieve all needed information, similar considerations can be done for wireless access technologies: it will probably be the integration of more than one wireless access technology that will lead to future automated and connected vehicles. For example, in highways or crowded cities, where the number of neighbors can be high, classical RF-based communications can be affected by severe packet collisions that increase delay and reduce communication reliability, and VLC could complement RF technologies to cover the performance gap [10]. This can decisive for a whole range of applications such as platooning [11] , cooperative adaptive cruise control, emergency warning, traffic light optimal speed advisory, etc., [6,12,13,14,15,16].

However, the performance of vehicular VLC networks, either when VLC is adopted alone or together with other wireless communication technologies still needs further investigations.

Given these considerations, in this paper we focus on how VLC can supplement crowd sensing and which is its role to achieve the vision of driving automation. Specifically, in this work we aim at evaluating the impact of VLC in vehicular crowd sensing applications to deliver the data, sensed and collected on board to the RSUs, assumed integrated in the traffic lights. Specifically, in this paper, we face the following questions:

- How can VLC support sensor sharing?
- Can the use of VLC improve the rate of data delivered at the RSUs when adopted in addition to other RF technologies?
- Which is the impact of number and position of RSUs?
- Can typical routing algorithms impact differently varying the field of view (FOV) of the receivers?

The paper is organized as follows: in Section 2, the main characteristics of VLC, the state of the art of vehicular visible light network (VVLNs) and the related standard are introduced; in Section 3, the addressed application and the considered scenario are presented; in Section 4 simulation tools and settings are described and in Section 5 results showing the impact of the use of sole VLC in terms of delivery rate are shown and compared with the case of VLC adopted together with IEEE 802.11p. Finally, in Section 6 our discussions and conclusions are drawn.

## 2. Related Works and Reference Standard

VLC represents an alternative and a complementary technology to the classical RF wireless communications. The interest in this kind of communication has recently intensified and this is mainly due to the following reasons:

- LEDs are more and more diffused since cheap and energy efficient;
- VLC uses the visible light spectrum (380–780 nm) offering (about a 1000 times) greater bandwidth compared to the RF bandwidth;
- the visible light spectrum is unlicensed;
- VLC is safe for the human health;
- VLC does not interfere with electromagnetic devices (hence, it can be used also in hospitals or airplanes);
- the limited penetration capabilities allow higher reuse factors and lower interference from neighbors.

Beside these advantages, it has to be remarked that VLC is susceptible to ambient light and weather conditions, it covers limited distances and does not cross obstacles [8,9].

The high reliability and efficiency of LEDs made this kind of lights widely used in vehicle headlights, turn signals, taillight and stop lights. Given also their lifetime, LEDs are diffused in the new city lights and in traffic lights as well. However, in spite the numerous advantages, VLC systems have been investigated in vehicular scenarios only recently.

The several advantages of VLC systems over RF systems and some potential algorithms to jointly manage the two technologies, have been already surveyed and demonstrated in indoor scenarios [17]. In [18], for example, a new protocol to improve horizontal and vertical handover mechanisms in mobile environments is proposed to keep VLC connections stable whenever a user moves indoor among different hotspots and to operate a vertical handover whenever the device moves on the edge between VLC and another system. In [19], authors proposed an indoor hybrid system that integrates WiFi and VLC through an handover mechanism that dynamically distributes resources.

Differently from indoor scenarios, in outdoor vehicular networks, challenging tasks still need to be faced [10].

Some outdoor experiments successfully demonstrated data rate of the order of 1 Gb/s over hundred meters [20] and others are concentrating on the comparison between typical RF channels and optical ones in vehicular applications addressing the unique capabilities and limitations of the last ones [21]. In [13], the impact on V2V communications of realistic headlamp beam, road reflected light and photodiode (PD) position in the car is evaluated, showing how the height of the receiving PD can improve or decrease the performance. In [22], a novel VLC receiver architecture designed for mobile multi-channel communications is presented. The proposed model is designed to be adopted for long distance communications and low signal-to-noise ratio (SNRs). Work in [23] presents the scheme for a new detector for multiple-input single-output (MISO) outdoor VLC systems together with an optimal power allocation scheme, and the bit error performance is derived.

To better understand which kind of receivers are most suitable for automotive application [24] presents a survey on recent solutions provided by the existing literature. In addition, authors propose a further idea to enhance the receivers in order to become more compatible to the requirements imposed by vehicular applications.

The adoption of VLC as a complementary technology for the internet of vehicles has been considered in [9], where a congestion-adaptive algorithm was proposed to jointly manage VLC and another RF technology to improve the performance of the network in terms of packets delivered and delay.

The increasing interest in this kind of communication is also demonstrated by the development of the IEEE 802.15.7 standard, which defines the physical (PHY) and medium access control (MAC) layers for the visible light spectrum and allows to support audio and video services also in mobile visible links [25].

IEEE 802.15.7 defines three different PHY layers, that differ for the adopted modulation and coding scheme, as shown in Table 1. Independently on the adopted physical layer, the standard adopts different types of forward error correction (FEC) for channel coding: for outdoor applications, where distances may be long and sunlight or public illumination may interfere, short data frames obtained with concatenated Reed-Solomon (RS) and convolutional code (CCs) are preferred [26].

At the MAC layer, the IEEE 802.15.7 standard foresees the use of carrier sensing multiple access with collision avoidance (CSMA/CA): each node transmits only if the medium is sensed as idle after a random backoff interval time; then, the message is considered correctly received only upon the reception of an acknowledgment at the transmitter.

Since FEC codes are made available by PHY I and PHY II [25], in this work we consider PHY I with variable pulse-position modulation (VPPM), optical rate equal to 400 kHz, RS code, and data rate $R_{V}=266.6$ kb/s.

However, it has to be highlighted that even though the standard identifies vehicular networks as a potential application area, it does not provide any specific implementing rules regarding V2V or V2I. Hence, many developers in the field do not refer to the IEEE 802.15.7 when developing their prototypes. Given this context, a revision of the standard, known as IEEE 802.15.7r1, is under study, addressing vehicular communications as a fundamental VLC use case. In this case, the standard considers the requirements of vehicular communications and aims to enhance mobility, data rates, robustness and the networking protocols [10]. To meet also this aspect and considering that some experimentations reached data rate of tens Mb/s, in this work we also consider VLC at 10 Mb/s.

## 3. Reference Application and Scenario

We address vehicular crowd sensing applications, where vehicles cooperate to sense and collect urban data requested by data centers [27]. Specifically, we assume that each vehicle periodically generates a packet containing some information collected on board, such as the vehicle identification, position, speed, direction and acceleration. Both IEEE 802.11p and VLC are assumed integrated on board and can be used separately or jointly as will be better detailed in Section 4.

Independently on the wireless interface, each vehicle attempts to transmit its data directly to the nearer RSU that will be in charge to forward them to a remote data center; if a direct transmission is not possible (due, for example, to distance), multi hop communication is exploited. The RSUs are assumed positioned at the crossroads; specifically, RSUs for VLC can be considered integrated in the traffic lights or road lamps, thus avoiding expensive installation, whereas RSUs for IEEE 802.11p need to be set up when necessary.

**Figure 1:**
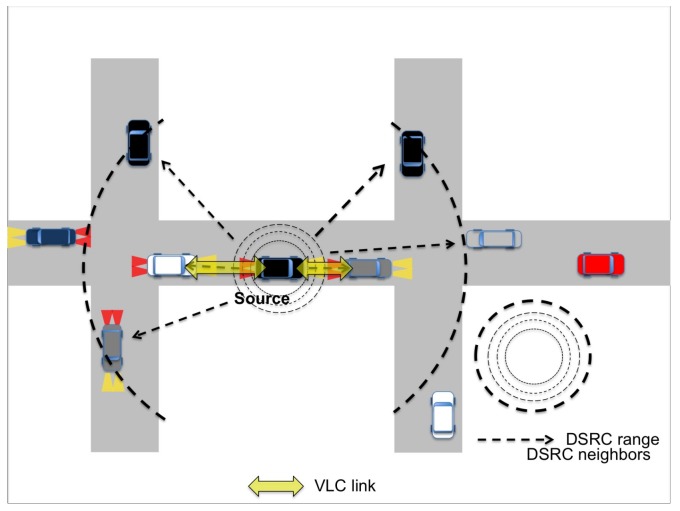
VLC and IEEE 802.11p links and coverage.

When VLC is addressed, transmissions happen through the head or rear LED lights, whereas receptions are typically left to photodiodes, assumed integrated in the vehicles lights themselves, as depicted in Figure 1. Reception could be performed also through the use of image sensors, which are devices that produce electrical image signal converted from reading object information in the form of light, whereas PDs convert light into electrical voltage levels from its surface area. Image sensors offer a wider detection area by using a wider angle sensor [28]. However, image sensors would incur high cost, high power consumption and low processing speed, whereas PDs are low cost, low power consumption and provide faster processing speed [29]. This is the reason why we consider PDs in this work.

The transmitter (LED) is characterized by a certain angle of irradiance and the receiver (photodiode) by its FOV: wider angles provide larger service areas, but also lead to performance degradation because of higher probability of receiving undesired light interference.

A transmission between two vehicles occurs if and only if (1) the virtual line connecting them is inside the transmitter angle of irradiance and the receiver FOV; (2) the virtual line connecting them does not cross any other vehicle or building; (3) the received power $P_{r}$ is higher than the receiver sensitivity $P_{r_{min}}$ and (4) the signal to noise and interference ratio (SINR) is higher than a threshold $\gamma_{\min}$, where the SINR can be evaluated as [9,30,31,32].

(1) $$\mathrm{SINR}=\frac{\beta^{2}P_{r}^{2}}{I+\sigma_{\mathrm{shot}}^{2}+\sigma_{\mathrm{thermal}}^{2}}$$

with β the detector responsivity, *I* the interference power, $\sigma_{\mathrm{shot}}^{2}$ the shot noise variance given by all the background light sources, such as sunlight and other artificial lights and $\sigma_{\mathrm{thermal}}^{2}$ is the thermal noise variance, both assumed Gaussian distributed [33]. The received power $P_{r}$ is evaluated as

(2) $$P_{r}=H\left(d,\theta ,\psi \right)P_{t}$$

where $P_{t}$ is the transmitted power and H(d,θ,ψ) represents the DC channel gain, which follows the generalized Lambertian model [34].

$$H\left(d,\theta ,\psi \right)=\left\{\begin{array}{cc} \frac{(m+1)A}{2\pi d^{2}}\cos^{m}\left(\theta \right)\cos\left(\psi \right) & \mathrm{if}\psi <\Psi_{C}\\ 0 & \mathrm{otherwise} \end{array}\right.$$

where A is the physical area of the detector, d is the distance between the transmitter and the receiver, θ is the angle of irradiance at the transmitter, ψ is the angle of incidence at the receiver, $\Psi_{C}$ is the half width of the FOV at the receiver and m represents the order of the generalized Lambertian radiant intensity and it is given by $m=-\ln2/\ln(\cos\phi_{\frac{1}{2}})$ where $\phi_{\frac{1}{2}}$ is the half power angle at the transmitter. A synthesis of the main parameters and their meaning can be found in Table 1. As can be observed, the adopted parameters are coherent with the European rules on vehicles LED lights [35].

## 4. Evaluation Tools and Settings

**Figure 2:**
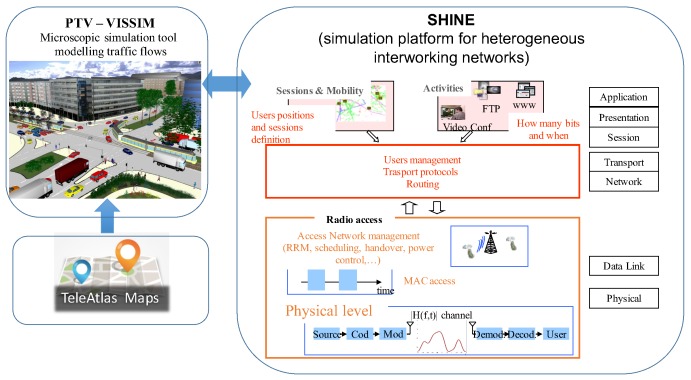
Integrated simulation platform for traffic and network performance evaluation.

Results are obtained in realistic vehicular scenarios by using an integrated simulation platform, as illustrated in Figure 2: from the one hand, we produced realistic traffic traces through a micro traffic simulation tool called VISSIM, which allows to take into account the roads with their number of lanes, to reproduce car-following and lane changing, one way roads, speed limits, queues at traffic lights and vehicles acceleration and deceleration; on the other hand, we reproduced the characteristics of the whole protocol pillar for both IEEE 802.11p and IEEE 802.15.7 by using the simulation platform for heterogeneous interworking networks (SHINE) [36,37,38].

Specifically, VISSIM is a microscopic simulation software to model transit and traffic flow in urban areas as well as interurban motorways. It uses a psycho-physical car following model for longitudinal vehicles movement and a rule-based algorithm for lateral movements. VISSIM consists of different programs, including the traffic simulator, a microscopic traffic flow simulation model, and a signal state generator. Car-following and lane changing are at the basis of the traffic flow model of VISSIM. Using dynamic assignment and origin/destination (O/D) matrices it is possible to realize a simulation without defining single routes. More information on the model can be found in [38].

SHINE reproduces the behavior of interworking access-networks, taking care of all aspects related to every single protocol level of each access technology affecting the achieved performance. Its realization was planned to overcome the limitations of off-the-shelf network simulation tools in terms of capability to simulate the behavior of heterogeneous networks operating simultaneously and exchanging users. SHINE is based on a client-server structure and is constituted by one server-core simulator (Upper Layers Simulator, ULS) and a client simulator for each access technology considered (Lower Layers Simulators, LLSs). All aspects related to the access technologies adopted, hence related to the data-link and physical layers, are managed by the LLSs which are the client simulators and are specific for each access technology: IEEE 802.11p and IEEE 802.15.7 in this paper.

### 4.1. Application and Road Scenario

We focus on crowd sensing, which today is a popular application to provide different types of information with high capillarity in time and space. In this work, we assume that each OBU acquires from on-board sensors several basic vehicle parameters such as vehicle identification, position, speed and acceleration, that are packed into B=100 byte packets every $T_{s}$ seconds, hence with a data generation rate $\lambda =1/T_{s}$ packets/s. Packets are stored in the vehicle queue and then attempted to be delivered to the most suitable RSU through single or multi-hop communication.

**Figure 3:**
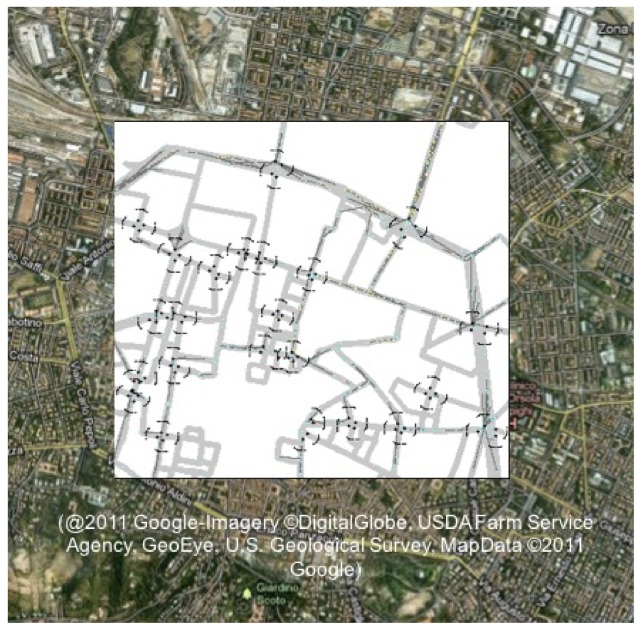
Simulated scenario: Bologna (Italy) downtown with up to 23 crossroads equipped with VLC and/or IEEE 802.11p RSUs. In each crossroad, black dots indicate the position of VLC RSUs, corresponding to the position of the traffic lights. The northern dot represents also the position of IEEE 802.11p RSU. Waves indicate the coverage and FOV of VLC RSUs.

Two scenarios, with fluent and congested traffic, are considered. The road-network layout of the scenario is plotted in Figure 3 and consists of a portion of the medium sized Italian city of Bologna of 1.8 × 1.6 ${\mathrm{km}}^{2}$. Specifically, in an area of 2.88 ${\mathrm{km}}^{2}$, 455 vehicles or 670 vehicles travel on average in the fluent and congested scenario, respectively.

### 4.2. Road Side Units (RSUs)

A variable number of RSUs are placed at some intersections of the scenario. Specifically, the following cases are considered:

1. 1-DSRC: 1 intersection equipped with 1 DSRC RSU (no VLC RSUs);
2. 1-VLC: 1 intersection equipped with 4 traffic lights with VLC capability acting as RSU (no DSRC RSUs);
3. 1-DSRC & 1-VLC: 1 intersection equipped with 1 DSRC RSU and 4 traffic lights with VLC capability acting as RSU;
4. 23-DSRC: 23 intersections, each one equipped with one DSRC RSU (no VLC RSUs);
5. 23-VLC: 23 intersections, each one equipped with four traffic lights with VLC capability acting as RSU (92 traffic lights and no DSRC RSUs);
6. 23-VLC & 23-DSRC: 23 intersections equipped with both one DSRC RSU each and 4 traffic lights with VLC capability acting as RSU (92 traffic lights and 23 DSRC RSUs);

The four traffic lights are placed on the four directions of the considered junctions of the scenario; for each considered intersection, the RSU of IEEE 802.11p is placed in the same position as the northern traffic light of the four VLC RSUs.

When only one RSU is considered for a given technology, it is positioned in the most travelled intersection of the scenario.

### 4.3. Relay Selection Procedure

We assume vehicles equipped with both VLC and DSRC technologies, where DSRC indicates the use of IEEE 802.11p communication protocol, whereas RSUs can be installed for one technology only or for both. To verify the impact of the use of VLC in vehicular networks, we compare the following main approaches:

1. VLC only: the sole VLC is used to communicate and reach the RSUs;
2. VLC first: VLC is used anytime it is possible in order to maximally offload the DSRC network;
3. DSRC only: the sole DSRC is used to communicate and reach the RSUs;
4. DSRC first: VLC first is used only in those cases where DSRC is not possible,

Comparing the considered approaches, we can verify the performance of a sole VLC vehicular network with that of VLC used to offload DSRC. In fact, if from one side, VLC suffers of short ranges and limited visibility, on the other side it offers a large unlicensed bandwidth, reduced deployment costs and high spatial reuse, which makes the full bandwidth being used in almost all links.

### 4.4. Performance Indicator

The system performance is evaluated in terms of delivery rate $D_{R}$, which is the ratio of packets delivered to the RSUs through single or multi hop communications

(3) $$D_{R}≜\frac{\varphi_{RSU}}{\varphi_{gen}}$$

where $\varphi_{gen}$ is the overall number of packets generated, and $\varphi_{RSU}$ is the number of packets delivered to the RSUs.

***PHY and MAC layers.*** When V2V and V2I communications are carried by means of IEEE 802.11p, we assume a received power inversely proportional to the distance raised to the power of 2.75 and buildings obstructing the communications. Such a model, also adopted in [39], is well suited to the characterization of the IEEE 802.11p physical layer performance in real environments, as shown in [40] where measurements are reported. With the considered settings, listed in Table 2, the maximum range is 200 m. Sensing and random access procedures, with collisions and retransmissions, are reproduced in details, also including hidden terminals, exposed terminals, and capture effects. The most reliable mode is used, thus the nominal bit rate is 3 Mb/s.

When VLC is adopted, we assume a received power inversely proportional to the distance raised to the power of four and any obstacle obstructing the communications [33]. The angle of incidence of the transmitters are assumed equal to the FOV of the receivers, which, in turn, can be equal to 30° or 60°. With the considered settings, listed in Table 2, the maximum range is 50 m [9]. Also in the case of VLC, sensing and random access procedures, with all the consequences (even if less relevant in this case due to the reduced number of neighbors), are reproduced in details. Two possible data rate are considered: (i) 266.6 kb/s following IEEE 802.15.7 specifications and (ii) 10 Mb/s thinking to future on board installations.

***Routing.*** Each OBU attempts to forward its packets to the nearest RSU adopting a greedy forwarding (GF) routing algorithm [39,41]. Specifically, if a vehicle is under coverage of an RSU, it performs a direct transmission, otherwise it follows these steps:

- it selects the nearest RSU;
- it considers as possible relays the neighbors that are closer than itself to the destination;
- it reduces the number of available relays by considering only those within the FOV of the RSU;
- among the remaining relays, it considers the one nearest to the RSU and transmits its data. If no relay satisfy the conditions, the packets are stored in a local queue.

To keep a certain data freshness, if after a parametric time out (here set to 700 s) or after a maximum number of packets in queue (here set to 2000), no direct transmission (nor single hop neither multi hop) is available, the vehicle is supposed to transmit its data through the cellular network.

## 5. Results

Results show the delivery rate as a function of the packet generation rate for the two considered scenarios of fluent and congested traffic. If not specified, we assume a FOV of both vehicles and RSUs equal to 15°.

**Figure 4:**
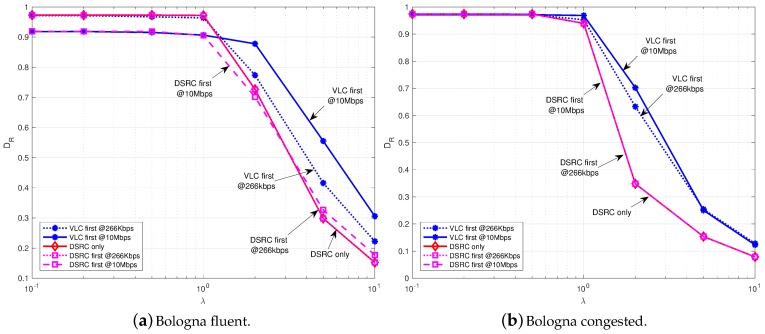
Delivery rate vs. packet generation rate with 1-DSRC varying the data rate of VLC in fluent (**a**) and congested (**b**) traffic conditions.

**Figure 5:**
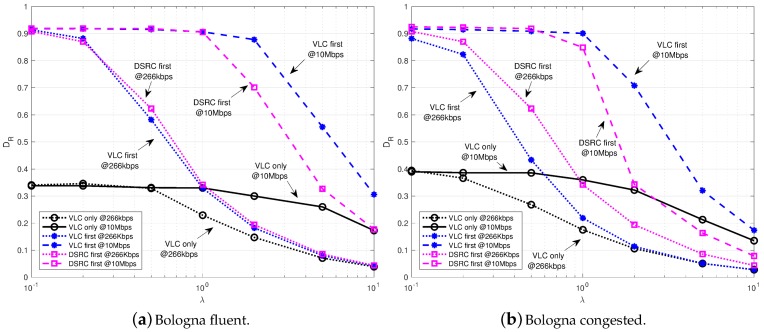
Delivery rate vs. packet generation rate with 1-VLC varying the data rate of VLC in fluent (**a**) and congested (**b**) traffic conditions.

Figure 4 and Figure 5 refer to a single crossroad equipped with RSUs of a single technology: 1-DSRC in Figure 4 and 1-VLC in Figure 5. This means that, V2V communications can be performed with DSRC or VLC, whereas, the RSU can be reached only with the technology it is equipped with.

Figure 4 refers to the case of 1-DSRC, hence a single crossroad is equipped with an RSU and it communicates thorough IEEE 802.11p. It can be observed how the delivery rate starts from a value near to 1 (all packets delivered) when the amount of data generated is small (λ≤1 packets/s) and then it reduces to less then 0.2 when the load is high (λ = 10 packets/s). It can be highlighted that, the performance of *DSRC first* is similar to that of *DSRC only* in fluent traffic conditions Figure 5a and just the same in congested traffic conditions Figure 5b, meaning that, due to the wider coverage provided by DSRC, the addition of VLC is almost negligible if DSRC is selected first. When VLC is selected first, for values of λ greater than 1 packets/s, $D_{R}$ is instead higher than both the *DSRC only* and *DSRC first* cases, demonstrating the effectiveness of VLC to increase the available resources. It can be also observed the impact of VLC data rate on $D_{R}$: in Figure 5a, when the data rate of VLC increases from 266 kb/s to 10 Mb/s, the $D_{R}$ increases of 15% for λ greater than 1 packets/s. The impact of the VLC data rate is lower in Figure 5b, where, instead, the important increase in $D_{R}$ (even over the 30%) is provided by the adoption of VLC with respect to *DSRC first*. In this case, in fact, given the higher number of vehicles, the IEEE 802.11p network is prone to collisions and the introduction of a new radio improves the performance.

On the opposite, Figure 5 refers to the case of 1-VLC, hence a single crossroad is equipped with four RSUs and it communicates thorough IEEE 802.15.7. As observable, any strategy allowing the use of the heterogeneous VLC and DSRC resources improves $D_{R}$ dramatically compared to the VLC only case. This is due to the lower connectivity level that is guaranteed by VLC in the vehicular network, since a link is formed only in line of sight and for shorter range with respect to DSRC. Comparing Figure 4 and Figure 5, it can be assert that, a smaller $D_{R}$ is obtained in the case of VLC RSUs with respect to DSRC RSU for the same λ; this was expected when VLC works at 266 kb/s, since it provides a smaller data rate compared to DSRC, but could surprise when VLC works at 10 Mb/s. We can explain this result thinking again to the smaller coverage range and the consequent low connectivity, which does not allow to reach the RSU in case of *VLC only*, independently on the data rate. In addition, the FOV of the receivers implies that vehicles must be almost in a line to communicate. This factor, added to the rules of routing algorithm, implies that an important percentage of vehicles cannot forward their messages to the RSU. On the other hand, we underline that the use of VLC has the great advantage to exploit the traffic lights that are already deployed on intersections; differently, DSRC RSUs require new installations.

**Figure 6:**
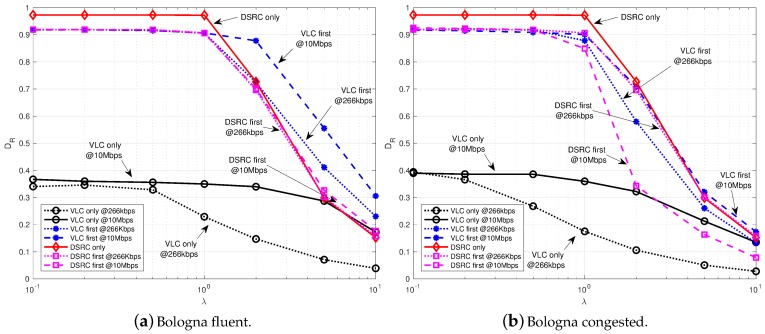
Delivery rate vs. packet generation rate with 1-DSRC and 1-VLC varying the data rate of VLC in fluent (**a**) and congested (**b**) traffic conditions.

To verify, now, the impact on the performance of the presence of both technologies at the RSUs, in Figure 6, 1-VLC & 1-DSRC are positioned at the most crowded junction of the scenario, near the central station of Bologna. If we compare this cases with those presented in Figure 4 and Figure 5, where a single technology was considered at the RSU, we can assert that, the joint use of DSRC and VLC at RSUs, considerably improves the performance of both *DSRC first* and *VLC* first. What can be underlined is that, when *VLC only* is adopted, $D_{R}$ is always lower than 0.4 even if VLC works at 10 Mb/s. As already explained, this is mainly due to the limited coverage range of VLC and the high directivity of lights, which reduce the performance when vehicles are not in a line.

**Figure 7:**
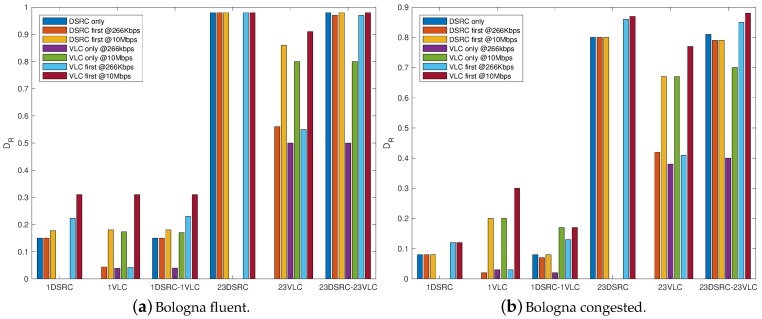
Delivery rate for various number of RSUs and λ=10 packet/s in fluent (**a**) and congested (**b**) traffic conditions.

The impact of different number of RSUs on the delivery rate is plotted in Figure 7, referred to λ=10 packtes/s. It is immediate to verify that when the 23 intersections are all equipped with RSUs, instead that a single one, $D_{R}$ largely increases. The better performance is achieved in the case of 23-DSRC and 23-DSRC & 23-VLC: this is mainly due to the presence of DSRC RSUs, with higher coverage with respect to VLC and with no problems due to directivity and line of sight. What could be not expected is that, also with 23 VLC RSUs, the maximum delivery rate is always lower than 0.7 when *VLC only* politic is adopted, also with 10 Mb/s.

**Figure 8:**
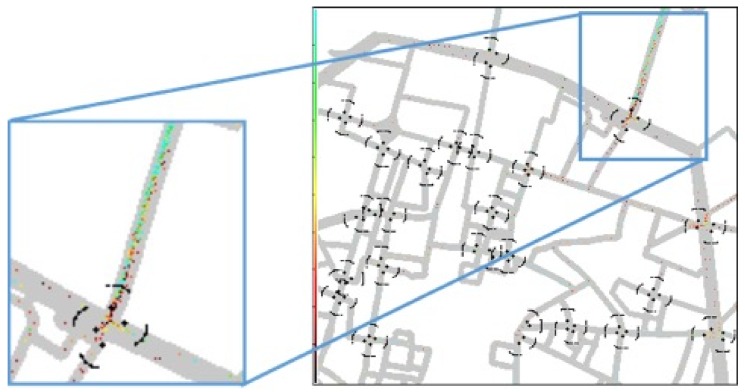
Congested traffic scenario with 23 VLC RSUs at 10 Mb/s. Colors of vehicles indicate the number of packets in the on board queue (light blue = no packets in the queue, black = full queue).

To better understand this aspect, Figure 8 shows, for the congested scenario with 23 VLC RSUs, the level of occupancy of the transmission buffer of each OBU in each position; darker colors mean higher number of packets stored in the vehicle queue (light blue means free queue, black means full queue). As can be observed, in spite of the fact that all intersections are equipped with VLC RSUs, vehicles cannot often empty their queue, even if, apparently, they can find neighbors to forward the packets. This is mainly due to two factors: (i) the FOV of both vehicles and RSUs is 15°, hence, the directivity is very high and if vehicles are not in line they can hardly communicate; (ii) the GF routing algorithm adopted to forward the packets through multi hop communication toward the nearest RSU, does not take into account the direction and FOV of vehicles.

**Figure 9:**
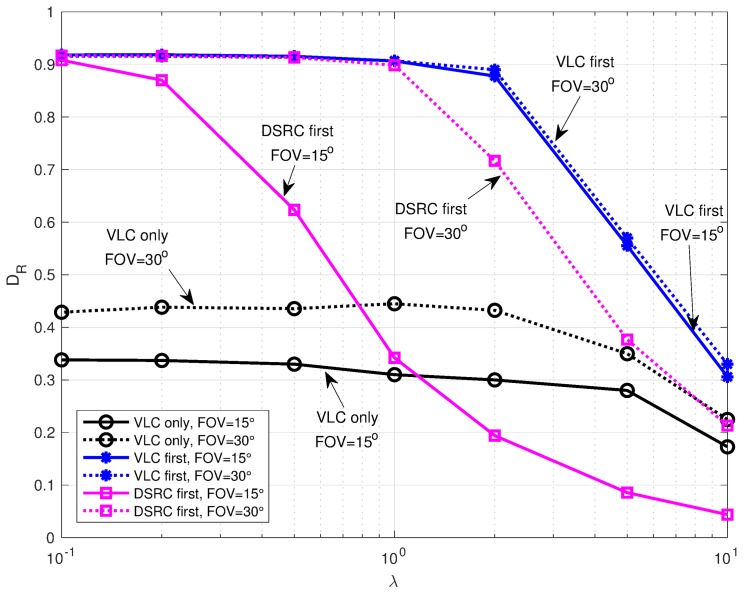
Delivery rate vs. packet generation rate in Bologna fluent scenario with 1-VLC at 10 Mb/s varying the FOV.

Hence, to also verify the impact of a larger FOV on the delivery rate, in Figure 9, we compared the performance of the different solutions for two values of FOV. and 30°. It can be observed, indeed, that, the adoption of a FOV = 30° instead that 15° , drastically improves the $D_{R}$ of both *VLC only* and *DSRC first*: when λ=1 packet/s, for example, the improvement is around 10% and 60%, respectively.

This also suggests further investigation on the joint impact of the routing algorithms and the FOV and the angle of irradiance of vehicles and infrastructures, taking into account also realistic heights of vehicles lamps.

## 6. Conclusions

We investigated, through realistic simulations in urban scenarios, the performance of a vehicular crowd sensing application, in terms of packets delivered to the RSUs. By considering vehicles equipped with both DSRC and VLC communication interfaces, we showed the delivery rate when the RSUs were equipped with only one of the two technologies or both. Results highlight the improvement of performance due to the use of VLC in addition to DSRC, especially in congested scenarios. The outputs also show the main limit of VLC, which is the reduced connectivity due to the high directivity of LEDs and PDs: if from the one hand, this imply lower interference, on the other hand, it provides less packets delivered to the RSUs.

Future investigations will be devoted to evaluate the joint impact of different routing algorithms and lights directivity on the performance. Additional studies could also consider the use of image sensors at the receiver side instead of PDs; since a major advantage of the image sensors is the ability to spatially separate multiple sources, in such case hence parallel data transmission and multiple VLC signal reception is possible. The use of image sensors would also allow to incorporate various image processing applications in VLC function, such as distance estimation of nearby vehicles, lane keeping application, or collision avoidance [29].

## Figures and Tables

**Table 1:** IEEE 802.15.7 physical layers.

	Modulation	FEC	Data Rate
PHY I	OOK or VPPM	no code or RS and/or CC	11.67 kb/s–266.6 kb/s
PHY II	OOK or VPPM	no code or RS	1.25 Mb/s–96 Mb/s
PHY III	CSK	no code or RS	12 Mb/s–96 Mb/s

**Table 2:** Parameters meaning and settings.

Parameter	Meaning	VLC	IEEE 802.11p
$P_{t}$	Transmission power	30 W	0.2 W
β	Detector responsivity	0.54 A/W	-
*A*	Physical area of the photodiode	1 ${\mathrm{cm}}^{2}$	-
$\psi_{c}$	FOV of the receiver	30°	-
		60°	
$\Phi_{\frac{1}{2}}$	Half-power angle of the transmitter	Equal to $\Psi_{C}/2$	
*m*	Order of the generalized	20 if $\Phi_{\frac{1}{2}}=15{}^{\circ}$	-
	Lambertian radiant intensity	5 if $\Phi_{\frac{1}{2}}=30{}^{\circ}$	
$\gamma_{\min}$	Minimum SNR	11.4 dB	10 dB
$d_{max}$	Maximum range	50 m	200 m
*R*	Data rate	266.6 kb/s	3 Mb/s
		10 Mb/s	
*B*	Packet size	100 bytes	
λ	Packet generation rate	[0.1–10] $s^{-1}$	
